# Insights gained from sequencing Australian non-invasive and invasive *Streptococcus pyogenes* isolates

**DOI:** 10.1099/mgen.0.001152

**Published:** 2024-01-10

**Authors:** Trent A.J. Butler, Chloe Story, Emily Green, Kirsten M. Williamson, Peter Newton, Frances Jenkins, Hemalatha Varadhan, Sebastiaan van Hal

**Affiliations:** ^1^​ Microbiology, NSW Health Pathology, John Hunter Hospital, New Lambton Heights, New South Wales, Australia; ^2^​ Microbiology, NSW Health Pathology, Wollongong Hospital, Wollongong, New South Wales, Australia; ^3^​ Hunter New England Population Health, Hunter New England Local Health District, Newcastle, New South Wales, Australia; ^4^​ Department of Infectious Diseases and Microbiology, NSW Health Pathology, Royal Prince Alfred Hospital, Sydney, New South Wales 2050, Australia; ^5^​ Central Clinical School, University of Sydney, Sydney, New South Wales 2006, Australia

**Keywords:** group A streptococcus

## Abstract

Epidemiological data have indicated that invasive infections caused by the Gram-positive cocci *Streptococcus pyogenes* (group A streptococcus, GAS) have increased in many Australian states over the past two decades. In July 2022, invasive GAS (iGAS) infections became nationally notifiable in Australia via public-health agencies. Surveillance for *S. pyogenes* infections has been sporadic within the state of New South Wales (NSW). This has led to a lack of genetic data on GAS strains in circulation, particularly for non-invasive infections, which are the leading cause of GAS’s burden on the Australian healthcare system. To address this gap, we used whole-genome sequencing to analyse the genomes of 318 *S*. *pyogenes* isolates collected within two geographical regions of NSW. Invasive isolates were collected in 2007–2017, whilst non-invasive isolates were collected in 2019–2021. We found that at least 66 different *emm*-types were associated with clinical disease within NSW. There was no evidence of any Australian-specific clones in circulation. The M1_UK_ variant of the *emm1* global pandemic clone (M1_global_) has been detected in our isolates from 2013 onwards. We detected antimicrobial-resistance genes (mainly *tetM*, *ermA* or *ermB* genes) in less than 10 % of our 318 isolates, which were more commonly associated with non-invasive infections. Superantigen virulence gene carriage was reasonably proportionate between non-invasive and invasive infection isolates. Our study adds rich data on the genetic makeup of historical *S. pyogenes* infections within Australia. Ongoing surveillance of invasive and non-invasive GAS infections within NSW by whole-genome sequencing is warranted to inform on outbreaks, antimicrobial resistance and vaccine coverage.

## Abbreviations

GAS, group A streptococcus; HNELHD, Hunter New England local health district; iGAS, invasive group A streptococcus; ISLHD, Illawarra Shoalhaven local health district; MLST, multilocus sequence typing; NCBI, National Center for Biotechnology Information; NSW, New South Wales; NSWHP, New South Wales Health Pathology; ST, sequence type.

## Impact Statement

Our study has described the molecular epidemiology of 136 non-invasive and 182 invasive *Streptococcus pyogenes* isolates collected from two communities in New South Wales (NSW), Australia. This work is vital as our healthcare system is encumbered by the breadth of clinical infection types and persistence of *S. pyogenes* within the Australian population. The results showed that at least 66 different *S. pyogenes* strains caused infections within NSW. There was substantial overlap in the *S. pyogenes* strains that caused non-invasive and invasive diseases. We found no evidence of a clone specific to the Australian continent. Notably, *emm-*types of global concern, including *emm*1, *emm*3.1, *emm*4*, emm*12, *emm*28 and *emm*89, were associated with most invasive infections, whilst many *emm-*types were sporadically detected. To our knowledge, we provide the first data on the *emm*1 global sub-lineage M1_UK_ within NSW. Fifty-eight per cent of our invasive disease isolates in 2017 were the M1_UK_ sub-lineage. This finding is important because other epidemiological studies have demonstrated that in 2017, there was a substantial increase in case numbers of invasive group A streptococcus (iGAS) diseases within the Hunter region of NSW, where these isolates were collected. We also found antimicrobial-resistance genes in less than 10 % of study isolates. These genes were mainly t*etM*, *ermA* or *ermB*. Since invasive *S. pyogenes* infections have become notifiable by public-health units in NSW in 2022, and prior studies on *S. pyogenes* within NSW are sparse, our data help to address critical public-health needs. It will be a valuable reference for future Australian studies investigating outbreaks or strain emergence, and for potential vaccination programmes.

## Data Summary

Raw sequence reads have been deposited into the National Center for Biotechnology Information (NCBI) database under BioProject PRJNA996294. Accession numbers for each isolate and their associated metadata are provided in Table S1, available with the online version of this article. All bioinformatics tools used for data analysis have been reported in Methods.

## Introduction

*Streptococcus pyogenes* (group A streptococcus, GAS) is a strict human pathogen with a wide variety of clinical manifestations, from self-limiting infections of the skin and throat causing impetigo and pharyngitis, respectively, to invasive infections including septicaemia, necrotizing fasciitis and streptococcal toxic shock syndrome [1]. The bacteria are transmitted from person to person, via droplet or direct contact from colonized or infected individuals. Specific M proteins located on the bacterial cell surface mediate adherence and internalization of GAS into epithelial cells [2, 3]. Additional pathogen virulence gene expression, as well as resistance to host immune mechanisms, may result in microbial entry into sterile sites (e.g. blood, bone, joint fluid or deep tissue) causing invasive group A streptococcus (iGAS) infection [4, 5]. Patient risk factors for developing iGAS include older age, immune disorders, diabetes and intravenous drug use [6].

GAS infections are strongly associated with post-infection immune-mediated diseases, including acute rheumatic fever, rheumatic heart disease and post-streptococcal glomerulonephritis [7]. These diseases represent autoimmune states involving cross-reactive antibodies and T cell dysregulation with repeated episodes of GAS infection, compounding tissue injury [1, 8]. Social determinants of health strongly influence the risk of developing GAS infection and subsequent immune sequelae [9]. Due to the preventable nature of antecedent infections, the overall incidence of immune-mediated complications is low in high-income countries [10]. However, First Nations peoples are still disproportionately affected; with an estimated incidence of approximately 374 cases of acute rheumatic fever per 100 000 persons per year in the Pacific and Indigenous Australia and New Zealand combined [10]. Although, the true overall incidence of GAS infection in Australia is unknown [7, 11, 12], the health impact is substantial with iGAS, rheumatic heart disease and GAS-associated kidney disease estimated to cost approximately AU$185.1 million (£97.4 million; £1=AU$1.9) and cause 110 deaths annually [13].

The incidence of GAS bacteraemia in the Hunter New England local health district (HNELHD) of New South Wales (NSW) has gradually increased over the last two decades, with a peak incidence of 9.00 cases per 100 000 population in 2017, which was double the previous 5 year mean (4.18 cases per 100 000 population per year for 2012–2016) [14]. Clinical cases of iGAS and/or laboratory isolation of GAS from sterile sites were made nationally notifiable through local Public Health Units in Australia in 2022 [15, 16]. This decision, in part, was made to better understand the dynamics of iGAS including the recent increases in Australia [7, 11, 12, 14], detect outbreaks and assess preventative strategies. Current control guidelines for iGAS within NSW include molecular typing for suspected outbreaks [16]. However, there remains a scarcity of reports on the molecular epidemiology of both invasive [17] and non-invasive GAS isolates found within NSW communities. Due to the dynamically changing number of GAS infections within NSW, updated reporting on strains in circulation is a public-health need. Whole-genome sequencing can be used to investigate GAS strains and obtain data on the carriage of genetic antimicrobial-resistance and virulence genes [18]. Furthermore, it is useful to monitor new infection sub-lineages occurring globally such as the recent M1_UK_ clone, which emerged from the parent global pandemic *emm*1 (M1_global_) clone [19]. To better understand GAS infections occurring within NSW and address public-health needs, we undertook sequencing of both invasive and non-invasive GAS isolates from two distinct hospital isolate collections within NSW.

## Methods

### Isolate collections

**Fig. 1.**
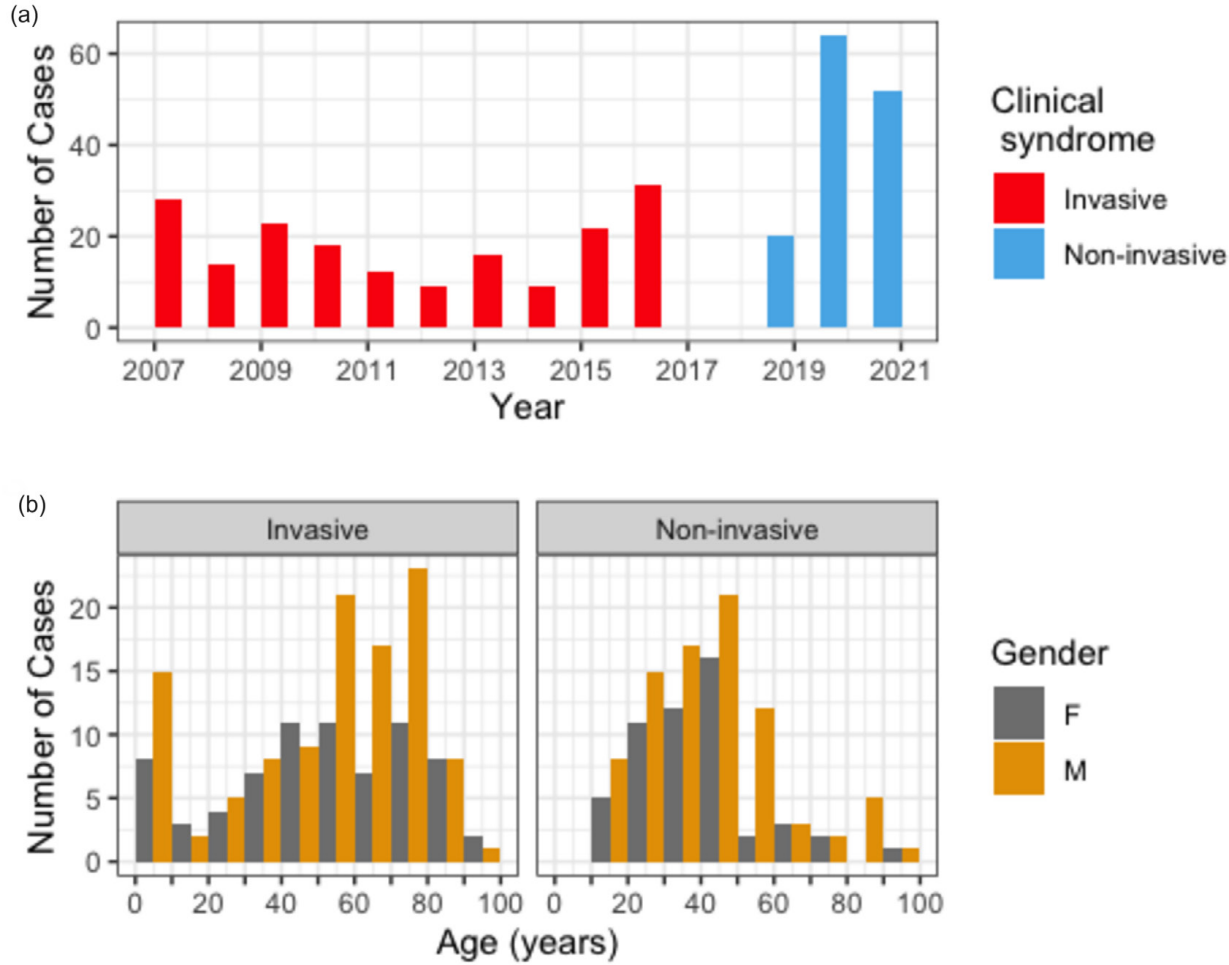
Epidemiology characteristics of sequenced isolates. (a) The distribution by year of collection. Invasive isolates were collected only in years 2007 to 2017 from the Greater Newcastle and Hunter regions of NSW and are shown in red. Non-invasive isolates were collected only in years 2019 to 2022 from the ISLHD and are shown in blue. Note that GAS isolates were not collected as part of this study during 2018. (b) The age distribution grouped by gender of patients presenting with *S. pyogenes* infection split by source of infection. F, Female; M, male. Note that non-invasive isolates were not collected from patients under 18 years of age.

A total of 318 group A streptococcal isolates from 318 patients (one isolate per patient) were retrieved for the study. Of these, 182 represented invasive isolates [sources include blood cultures (*n*=171), body fluids (*n*=10) and cerebrospinal fluid (*n*=1)] collected and stored between 2007 and 2017 (Fig. 1a), obtained from eleven public hospitals [serving a population of ~676 556 (2021)] within the Greater Newcastle and Hunter regions of the HNELHD. These hospitals were serviced by the NSW Health Pathology (NSWHP) John Hunter Hospital microbiology laboratory. Gender and age were known for 181 out of these 182 isolates. The remaining 136 isolates were obtained from patients presenting to Illawarra Shoalhaven local health district (ISLHD) hospitals [serving a population of ~404 000 people (2021)] with microbiology services performed at the NSWHP–Wollongong microbiology laboratory. These isolates represented non-invasive skin and throat isolates collected from patients presenting with cellulitis and pharyngitis, respectively, between 2019 and 2021 (Fig. 1a). Gender and age were known for all 136 of these isolates. Due to restrictions in the ethical approvals for this study, non-invasive isolates were collected only from people older than 18 years. Both laboratories are greater than 100 km from Metropolitan Sydney, cover metropolitan, inner regional and outer regional areas, service a lower socio-economic demographic, and have higher proportions of residents that identify as Aboriginal and/or Torres Strait Islander (5.9 % in HNEHLD and 3.5 % in ISLHD against a mean of 2.9 % across NSW census data) [20]. Non-overlapping time frames occurred as result of isolate collection criteria at each of the laboratories.

All bacterial isolates preserved in sterile nutrient broth with 15 % glycerol at −80 °C were rejuvenated following subculture and overnight incubation on horse blood agar in 5 % CO_2_ at 35 °C. GAS identity was confirmed using MALDI-TOF MS Biotypers (Bruker Daltronics) prior to DNA extraction.

### Genomic DNA extraction and whole-genome sequencing

DNA from each specimen was extracted following a lysis step. Extraction was undertaken for invasive isolates using Zymbiomics DNA microprep kits (Zymobiomics) and for non-invasive isolates using the Qiagen-EZ1 DSP virus kit on the Qiagen-EZ1 Advanced XL platform (Qiagen). Qubit high-sensitivity dsDNA fluorometry was used for DNA quantitation to ensure adequate quality of material post-extraction prior to library preparation. Whole-genome sequencing was performed on the Illumina MiSeq platform after paired-end libraries were prepared using the Illumina DNA prep kit.

Sequencing metrics were optimized to aim for a target sequencing depth of 100× and minimum Q30 score across >90 % obtained reads with quality visualization performed using FastQC v0.11.9 [21]. Contamination and species identification was performed using a *k*-mer based approach using Kraken2 v2.1.2 with exclusion of sequences with <90 % matching GAS isolates [22, 23].

### Global context and bioinformatic analysis

To put our isolates into a global context, the dataset (*n*=318) was augmented by including 117 Illumina sequences of group A streptococcal genomes with the raw reads downloaded from the National Center for Biotechnology Information (NCBI) Sequence Read Archive (SRA) [24] (see Table S1). These sequences represented isolates from a range of (i) invasive infections, (ii) *emm-*types including *emm*1 sub-lineages (i.e. M1_UK_ and M1_global_) and (iii) those representing several different geographical regions.

Raw reads were trimmed using fastp v0.22.0 [25] prior to generating assemblies using Spades v3.12.0 [26]. Multilocus sequence typing (MLST) was used to characterize sequence types (STs), and was performed using the PubMLST database [27]. Virulence gene(s) and antimicrobial-resistance gene(s) were detected on the contigs using ABRicate v1.0.1 [28] against the virulence factor database (VFDB) [29] and amrFinder v3.11.14, respectively [30]. Greater than 85 % identity and 90 % coverage were required for detection of genes using these tools. *emm-*typing was performed using emmtyper with blastn v2.12.0 [31], against the downloaded Centers for Disease Control and Prevention (CDC) *emm*-type specific database (https://ftp.cdc.gov/pub/infectious_diseases/biotech/tsemm/). The two *emm*-like gene homologues (*enn* and *mrp*) were filtered with EMM clustering designation based on previous functional grouping of types into 48 groups [32, 33].

The trimmed reads were mapped against the *S. pyogenes* type strain reference sequence (NCBI accession no. CP028841.1) [34] using bwa-mem v0.7.17-r1188 [35]. Resulting BAM files were sorted with SNPs detected using freebayes v1.3.5 [36]. Consensus genomes were subsequently determined using bcftools v1.14 ignoring indels [37].

Recombination was determined using Gubbins v3.3.10 [38] with default parameters. A maximum-likelihood phylogenetic tree was reconstructed using iq-tree2 from the masked SNP multifasta file [39]. The GTR+F+G4 model was used for tree reconstruction and 100 bootstrap trees were included. Tree visualization was performed using the web-based program iTOL, which allows for mapping epidemiological data to the phylogeny [40]. M1_UK_ variants were identified in the dataset by screening all invasive and non-invasive *emm1* isolate genomes for the 27 SNPs defined by Lynskey *et al.* [19].

## Results

### Epidemiology of isolates

The iGAS isolates sequenced from the HNELHD between 2007 and 2017 showed a bimodal age distribution; 12.7 % of isolates were from children under 10 years old (*n*=23), 26.5 % of isolates were from people 10–49 years of age (*n*=48), and 60.8 % of isolates were from adults 50 years of age and over (*n*=110). Overall, 109 cases occurred in males (60 %), and 72 cases occurred in females (40 %). Cases in males predominated after 50 years of age (70 out of 110 cases, 63.6 %) (Fig. 1b).

Of the 136 non-invasive GAS isolates sequenced from adults in ISLHD between 2019 and 2022, 27.9, 19.9 and 27.9 % of cases were in the 18–29 year (*n*=38), 30–39 year (*n*=27) and 40–49 year (*n*=38) age groups, respectively. Only 33 cases (24.3 %) were from adults aged 50 years and over (Fig. 1b). Overall, 84 cases occurred in males (62 %), and 52 cases occurred in females (38 %). Cases in males predominated across all age groups (range 55–73 %), and occurred to a greater extent in adults aged 50 years or over (24 out of 33 cases, 73 %).

### Distribution of *emm*-types and MLST characterization

A total of 53 *emm*-types and 57 different STs were detected in the 182 iGAS isolates. The top seven STs, which accounted for approximately 60 % of invasive isolates, were ST-28 (*n*=40); ST-52 (*n*=20); ST-101 (*n*=14); ST-15 (*n*=14); ST-36 (*n*=10); ST39 (*n*=7); ST-182 (*n*=5). A total of 33 *emm*-types and 34 different STs were detected in the 136 non-invasive isolates. The top seven STs, which accounted for approximately 61 % of non-invasive isolates, were ST-11 (*n*=22); ST-172 (*n*=22); ST-101 (*n*=14); ST-12 (*n*=6); ST-36 (*n*=6); ST-922 (*n*=6).

Across all 318 isolates, there were 66 *emm-*types and at least 75 different STs in total (Table S1). Seven *emm*-types represented 53.8 % of the entire database [*emm*1 (*n*=42), *emm*89.0 (*n*=30), *emm*53 (*n*=26), *emm*28 (*n*=24), *emm*59.1 (*n*=22), *emm3.*1 (*n*=14) and *emm1*2 (*n*=13)]. All isolates were able to be assigned an *emm-*type; however, three isolates were novel and unable to be matched to an existing ST. These have been assigned STs 1443–1445 by PubMLST. *emm*-types were not restricted to any one genomic backbone, with 16 out of the 66 *emm*-types linked to multiple STs.

**Fig. 2.**
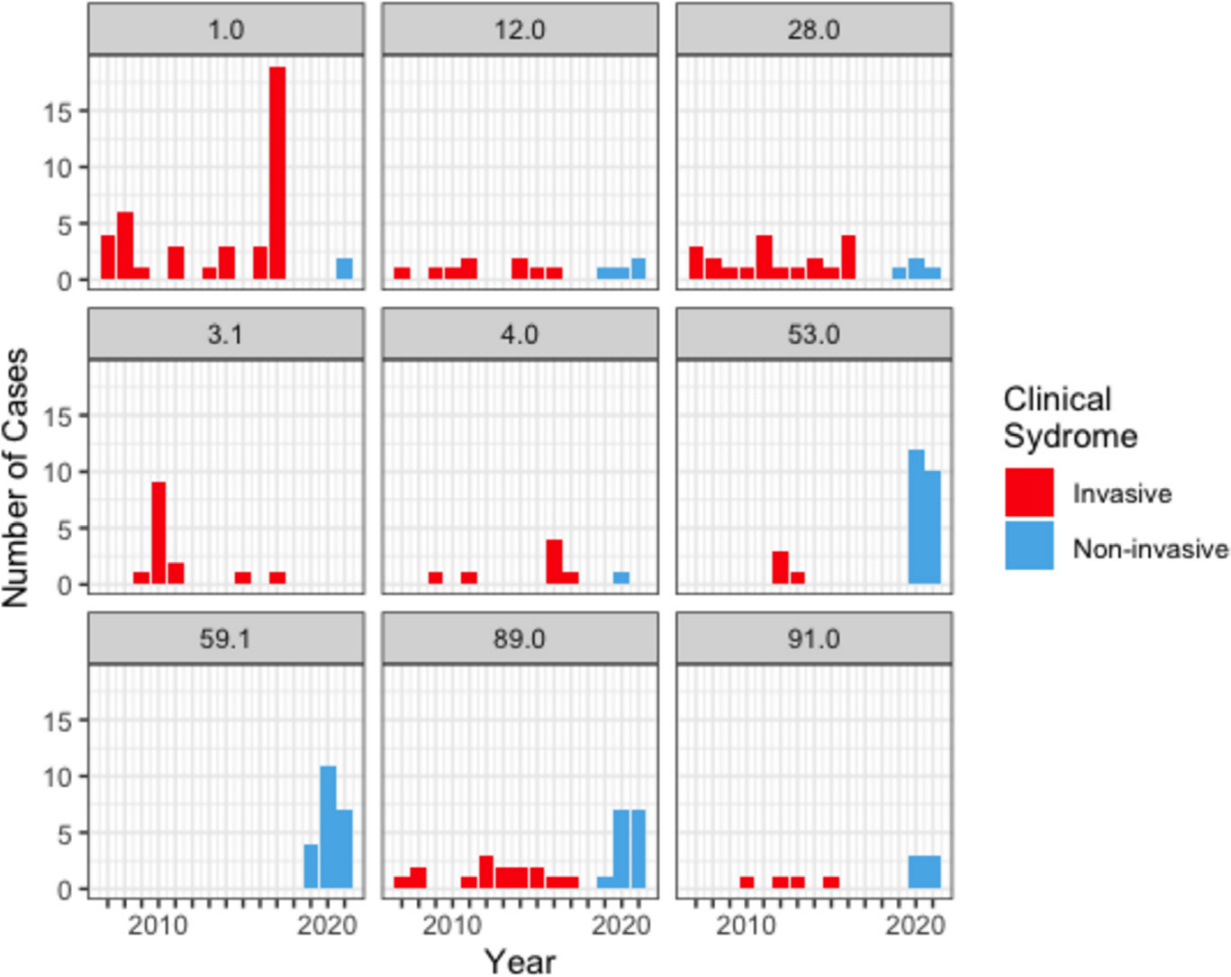
Histogram of sequenced *S. pyogenes* infection isolates grouped by *emm-*type and by year of isolation. Stacked columns are coloured by clinical syndrome presentation (invasive isolates are shown in red, and non-invasive isolates are shown in blue). To circumvent a potential sampling bias, the analysis was restricted to *emm-*types that represented ≥5 % of the dataset and occurred at least once in 3 or more years. Note that invasive isolates were collected only in years 2007 to 2017 from the HNELHD. Non-invasive isolates were collected only in years 2019 to 2022 from the ISLHD. GAS isolates were not collected as part of this study during 2018.

In the context of clinical presentations for the 318 isolates, 10 out of the 14 STs with at least five isolates caused both invasive and non-invasive infections (Table S1). To limit sampling bias, we restricted the analysis to those ST types that occurred at least once for 3 or more years and represented at least 5 % of the isolates in the dataset (*n*>15). We found that certain strains were more commonly detected in patients with invasive infection (e.g. *emm*1.0 ST-28 and *emm*28.0 ST-52) compared to non-invasive infections (e.g. *emm*53.0 ST-11 and *emm*59.1 ST-172) (Fig. 2).

### Phylogeny of GAS in NSW, Australia

**Fig. 3.**
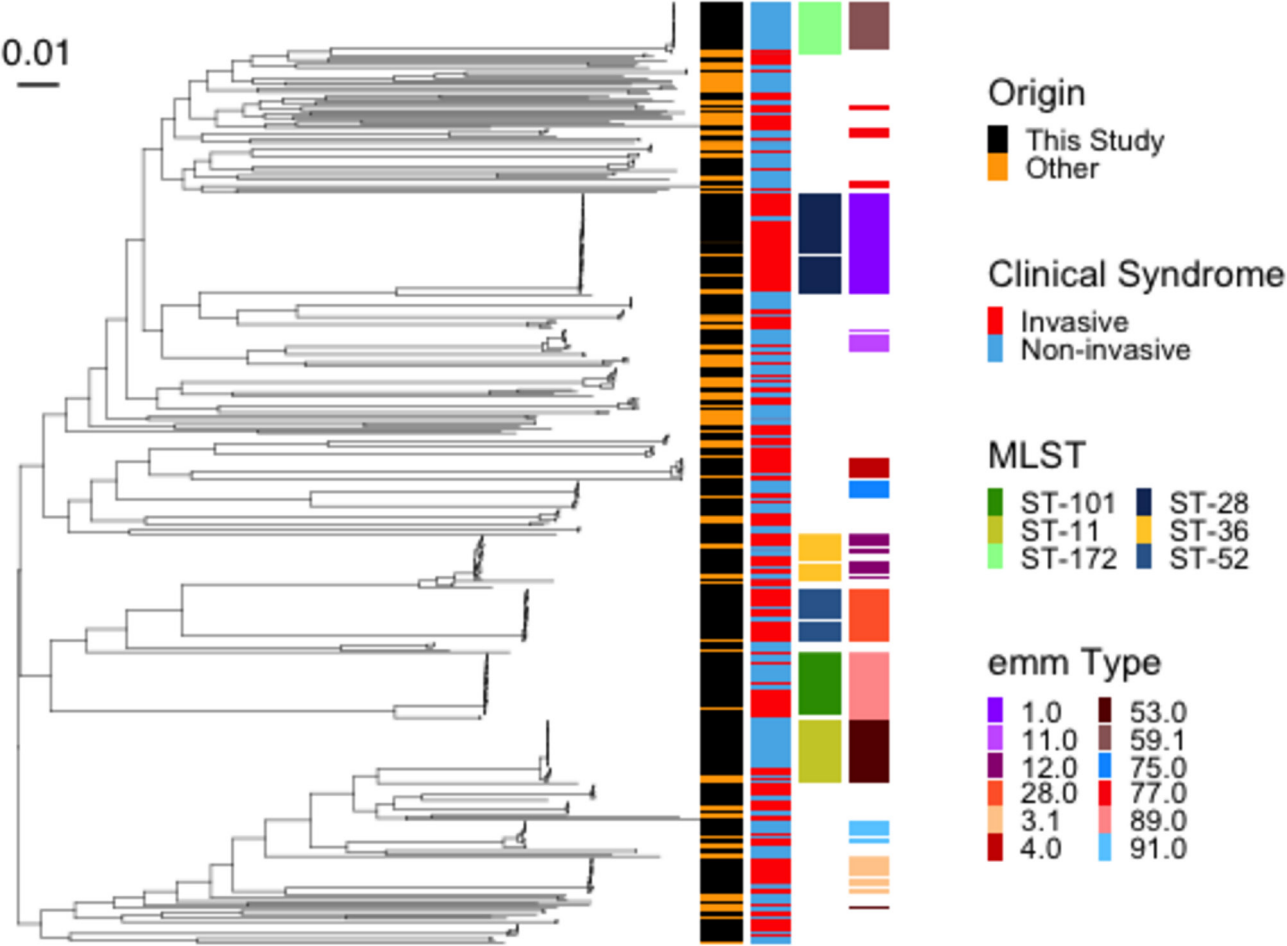
Maximum-likelihood phylogeny following masking of recombination of 438 *S. pyogenes* sequences. This included 117 internationally characterized sequences from invasive and non-invasive disease cases that were collected across nine countries. Associated metadata are depicted by coloured tiles next to the tree as indicated in the key. Only MLST and *emm*-types that represented ~5 % of the dataset and occurred in more than 1 year are shown. Scale bar shows phylogenetic distance.

We reconstructed a maximum-likelihood phylogenetic tree to understand better the interrelationships between GAS found in NSW, Australia, and those seen in other countries. To make this tree, we masked recombination sites and added 117 international characterized sequences from a diversity of *emm*-types to our 318 GAS sequences for a total of 435 sequences (Table S1). International sequences were spread across the phylogeny, and we found no evidence of an Australian-restricted clone. The tree demonstrated a population structure with a large genetic diversity with multiple *emm*-types. There were several clonal expansion events observed, with little diversity within the clones (Fig. 3). As expected from the analysis of *emm-*types and STs, invasive and non-invasive disease manifestations were spread across the phylogeny, with a considerable number of *emm*-types and STs causing both invasive and non-invasive infections.

In our study, the within *emm*-type genomic diversity of our 318 isolates increased as circulation time increased. For example, genetically identical isolates were common within *emm*59.1, which had a mean diversity of 5 (IQR (interquartile range) 1–8) SNPs across a 2 year time frame compared to *emm*1, where the mean SNP difference was 33 (IQR 18–47) from isolates representing a 14 year time frame.

### Characterization of *emm*1 sequences of M1_UK_ clone within NSW, Australia

**Fig. 4.**
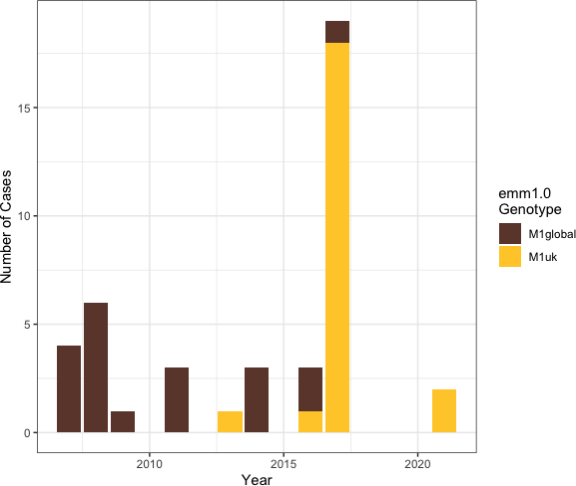
Characterization of our *emm1* isolates as M1_global_ or M1_UK_. Stacked bar graph of current study group A *S. pyogenes* infection isolates (*n*=42) grouped by year of isolation with columns coloured by *emm1* genotype. Note that comparisons from 2007 to 2017 only include iGAS isolates collected within the HNELHD, and comparisons from 2019 to 2021 only include non-invasive isolates collected within the ISLHD. GAS isolates were not collected as part of this study during 2018.

A total of 42 *emm*1 types were identified from invasive (*n*=40) and non-invasive (*n*=2) isolates. Based on 27 defining SNPs, these *emm*1 sequences were further characterized into M1_UK_ or M1_global_ clones. M1_global_ clones comprised all *emm*1 isolates collected before 2013. The first M1_UK_ clone was seen in our dataset in 2013, followed by a large expansion event almost replacing the M1_global_ clone in 2017 (Fig. 4).

### Antimicrobial-resistance genes and virulome

Antimicrobial resistance was more frequently encountered in non-invasive isolates than invasive isolates. Thirty-two (invasive, 10; non-invasive, 22; 10 % of total, *n*=318) isolates harboured the *tetM* gene associated with tetracycline resistance. Nineteen isolates (invasive, 4; non-invasive, 15; 6 % of total, *n=*318) harboured *ermA* or *erm*B genes, and one isolate (<1 % of total, *n*=318) harboured *mefA* genes, which are all associated with macrolide resistance. Two isolates (<1 % of total, *n*=318) harboured *dfrG* associated with sulfamethoxazole resistance. DNA gyrase mutations were not detected in any isolate.

Although numerous virulence genes have been associated with GAS, we restricted the analysis to only the 11 chromosomal and phage-associated superantigens present in the virulence factor database that was used. A minimum of one and maximum of six of these superantigen genes were detected in each isolate in our collection. Approximately 85 % of all of our isolates harboured between three and five of these superantigens. The proportions of the total number of superantigens detected remained similar when isolates were grouped by clinical presentation (Table S1).

## Discussion

Our study has described the molecular epidemiology of iGAS isolates collected over 11 years from the Hunter region and non-invasive GAS isolates collected over 3 years from the Illawarra region of NSW, Australia. Whilst the 66 different *emm-*types observed indicate that many GAS strains cause infections in these communities, a larger Australian dataset focused solely on iGAS cases from Victoria (*n*=1202; sampling period 2007–2017) found 140 *emm-*types [41]. Our findings regarding the most common *emm*-types for iGAS cases were similar to the Victorian study [41], and a study performed in the Western Sydney local health district, which found 27 *emm-*types (*n*=55; sampling period 2008 and 2010) [17]. Given the lack of evidence for Australian-specific clones, it is of little surprise that during the study period internationally significant strains (e.g. *emm1*, *emm3.1*, *emm12*, *emm28* and *emm*89) that make up the bulk of iGAS disease in Western countries also caused the majority of iGAS cases within these eastern Australian states [17, 41, 42].

Previous studies found that certain *emm*-types were more frequently associated with invasive infections [43–46]. This led to the concept of an invasive index, which estimates the propensity for a strain to be associated with invasive infection compared to non-invasive infection [46]. Our data are consistent with these prior studies, and nominally support the invasive index concept (Fig. 2). However, due to the non-overlapping periods and different sampling across the two different time periods, firm conclusions cannot be drawn on the association of certain *emm*-types with invasiveness

An increasing body of molecular evidence, including recent whole-genome sequencing data, has reinforced the concept that within each GAS *emm*-type, genetically similar clones cause both non-invasive and invasive infections; indeed, comparison of individual lineages by infection type has usually revealed that any genetic differences were minimal, and sometimes no genetic differences were found [45, 47–56]. For example, an *emm*1 outbreak lasting approximately 20 days that affected nine people (two invasive cases and seven non-invasive cases) associated with a maternity unit in the UK displayed zero SNP differences between all but one isolate [53]. Our data on within *emm*-type genomic diversity within NSW are similar to those from international studies, which have noted minimal divergence in clones over years [48, 50, 53, 57–63]. The genetic similarity indicates that many epidemiologically unrelated clones circulating within the community would be indistinguishable over short time frames thereby posing a false notion of an outbreak at a molecular level. Therefore, spatial and temporal relatedness in addition to ‘molecular’ similarity is important to confirm an outbreak.

In our study, non-invasive and invasive isolates of the same *emm-*type and ST clustered together in the phylogeny. Reports by Shea *et al.* [51] and Hoe *et al.* [47] indicate that invasive infections could be sporadic events that repeatedly arise from the circulating pool of clones. Whilst likely, whether the invasive clones in our study arose from the circulating pool of colonizing non-invasive isolates was unable to be definitively determined due to the non-overlapping time periods of this study. Recent studies from school classrooms within the UK and indigenous communities within the Northern Territory of Australia have demonstrated that asymptomatic throat carriage of temporary duration is a common reservoir for many GAS strains [64, 65]. Failure to investigate asymptomatic carriage is a limitation of our study. Future studies should better account for this and investigate whether asymptomatic carriage provides a pool of clones from which invasive infections can arise.

The M1_UK_ strain is of international concern as it produces more superantigen SpeA than its progenitor M1_global_ strains [19, 66]. SpeA is a virulence factor that prolongs survival against the immune system [67]. The oldest known M1_UK_ isolates were collected in the UK in 2010 [19]. By 2013, M1_UK_ was the UK’s most commonly sequenced *emm*1 clone [19, 68]. Recently, Davies *et al.* [66] showed that 50–75 % of sequenced *emm*1 isolates in Victoria and Queensland during 2017–2020 were M1_UK_ strains, and that it has been present in Australia since at least 2013. We had similar findings, including detection of M1_UK_ amongst the invasive isolates that were collected in 2013 within NSW. The notable expansion of M1_UK_ isolates in 2017 aligns with a significant peak in iGAS case numbers in the HNELHD during the same year [14], which also occurred in other states [41].

A key aim of our study was to understand the molecular epidemiology of invasive and non-invasive *S. pyogenes* isolates in NSW. Whole-genome sequencing incorporated into surveillance programmes could assist our public-health teams in outbreak investigation, specifically to exclude outbreaks. Whole-genome sequencing has advantages over *emm*-typing and MLST for outbreak investigations for GAS because small genetic differences can be used to ‘rule out’ links in appropriate epidemiological contexts [48, 50, 62]. Key findings from an Australian healthcare and economic modelling study revealed that non-invasive (and locally invasive infection) accounted for approximately 90 % of healthcare presentations for *S. pyogenes* infections and 80 % of healthcare-associated costs [13]. Public-health measures to reduce the burden of non-invasive infections, in addition to invasive infections, could positively impact on the overall healthcare costs [13, 69].

Finally, molecular epidemiology by whole-genome sequencing of invasive and non-invasive *S. pyogenes* isolates could provide rich information on vaccine coverage in our communities. The vaccine candidates undergoing clinical trials, such as the 30-valent vaccine, are the most promising tools to reduce GAS infection numbers [69–71]. On this note, under the assumption that all subtype *emm* alleles of a parent *emm-*type were covered by the 30-valent vaccine candidate [72], approximately 83 % of our invasive isolates and 61 % of our non-invasive isolates were covered or had documented evidence of cross-reactivity [70, 72]. With the inclusion of iGAS in the list of Public Health notifiable conditions and embedding whole-genome sequencing in continuous genomic surveillance, there will be a greater ability to track outbreaks, and inform on vaccine coverage.

## Supplementary Data

Supplementary material 1
